# The compliance of head-mounted industrial PPE by using deep learning object detectors

**DOI:** 10.1038/s41598-022-20282-9

**Published:** 2022-09-29

**Authors:** Velibor Isailovic, Aleksandar Peulic, Marko Djapan, Marija Savkovic, Arso M. Vukicevic

**Affiliations:** 1 Faculty of Engineering, University of Kragujevac, Sestre Janjic 6, Kragujevac, Republic of Serbia; 2 Faculty of Geography, University of Belgrade, Studentski trg 3/III, 11000 Belgrade, Republic of Serbia

**Keywords:** Computer science, Information technology, Software

## Abstract

The compliance of industrial personal protective equipment (PPE) still represents a challenging problem considering size of industrial halls and number of employees that operate within them. Since there is a high variability of PPE types/designs that could be used for protecting various body parts and physiological functions, this study was focused on assessing the use of computer vision algorithms to automate the compliance of head-mounted PPE. As a solution, we propose a pipeline that couples the head ROI estimation with the PPE detection. Compared to alternative approaches, it excludes false positive cases while it largely speeds up data collection and labeling. A comprehensive dataset was created by merging public datasets PictorPPE and Roboflow with author’s collected images, containing twelve different types of PPE was used for the development and assessment of three deep learning architectures (Faster R-CNN, MobileNetV2-SSD and YOLOv5)—which in literature were studied only separately. The obtained results indicated that various deep learning architectures reached different performances for the compliance of various PPE types—while the YOLOv5 slightly outperformed considered alternatives (precision 0.920 ± 0.147, and recall 0.611 ± 0.287). It is concluded that further studies on the topic should invest more effort into assessing various deep learning architectures in order to objectively find the optimal ones for the compliance of a particular PPE type. Considering the present technological and data privacy barriers, the proposed solution may be applicable for the PPE compliance at certain checkpoints where employees can confirm their identity.

## Introduction

The ongoing technological progress has significantly increased reliability of industrial equipment—which has left human factors as the leading cause of workplace accidents. Reports from the US alone indicate that injury costs were estimated to $161B, while an average annual cost was $1100 per worker (i.e. a factory of 1000 workers has an annual cost of $1.1 M due to injuries and accidents)^1^. Occupational safety and health (OSH) is an interdisciplinary scientific field that aims in creating a workplace environment that ensures employees well-being, safety and health at work^2^. Together with employee education, the frontline OSH measure for preventing workplace injuries is the use of personal protective equipment (PPE); which is also regulated with the corresponding standards and guidelines for each sector of industry. However, the practice has shown that the misuse of PPE represents a serious issue for both companies, healthcare systems budgets (i.e., 360B dollars annually to the US alone^3^), and employees that are facing consequences of occurred injuries. Finally, the reports show that large portions of recorded injuries could be prevented through the proper use of PPE^4^, which affordability nowadays do not represent an obstacle for ensuring workplace safety.

Safety managers in most companies have limited capacities for timely and objective observation of large manufacturing halls and hundreds of employees or visitors that circulate within workplaces. As an alternative, there is a tendency and growing need for the computerized tools that could assist safety managers by indicating violations from prescribed protection measures. First papers on this topic were focused on using sensors that were placed on the equipment itself^5^, or development of the conceptual framework of smart PPE compliance^6^. In the following paragraph, we will review recent studies on the topic of application of computer vision (CV) techniques for enabling contactless PPE compliance of head-mounted PPEs.

### Related studies

Chen and Demachi proposed a solution that uses OpenPose for the detection of body landmark points and YOLOv3 for PPE detection, while the PPE compliance was done by analyzing the geometric relationships of individual’s keypoints and detected PPE^8^. Balakreshnan et al., proposed a software architecture, which included an IoT module and the Microsoft Azure Custom Vision AI and Intelligent AI Services to assess detection of safety glasses in laboratory environments^9^. So far, the majority of related studies were focused on construction engineering—where the objective was to check if employees are using hardhats and yellow vests. Wu et al. assessed application of the SSD architecture for detecting hardhats of various colors on construction sites^10^. Delhi et. al used YOLOv3 to detect the presence of hardhat and safety jackets^11^. The same detector was recently assessed for the compliance of PPEs mounted on various body parts (i.e. hard hat, shirt, belt, gloves, pants, shoes)^12^. Zhafran et. al assessed the Fast R-CNN architecture for compliance of masks, gloves, hardhats, and vests—reporting the drop of accuracy with the increase of distance and variations of ambient light^13^. In addition, there are several studies as a result of the Covid19 pandemic crisis, which deal with the detection of protection masks. Loey et al. used YOLOv2 for the medical mask detection^14^, while a separate study combined the SSD detector with the MobileNetV2 for the compliance of medical masks used during the Covid19^15^. Zhang et al. have developed their own Depthwise Coordinate Attention (DWCA) algorithm based on YOLOv5 architecture for hardhat detection^16^.

As illustrated in Table 1, there is no comprehensive study on the topic of AI/CV-based PPE compliance—instead, there are separate studies focused on specific PPE types that were of interest for specific type of industry. Additionally, previous studies mainly considered a single architecture, which makes it difficult to perform direct comparison between studies since different datasets were used for the training. The purpose of our ongoing AI4WorklaceSafety (http://www.ai4workplacesafety.com (Accessed 29. 10. 2021)) initiative is to perform an integral study, which will envelop various types of PPE (used across different industries to protect different body parts/functions). In our previous study, we assessed an approach based on combining pose estimation algorithms for determination of region of interests—which need to be forwarded to a classifier^7^. We found that the compliance of a head-mounted PPE is a specific case—as there is a high variability of PPEs in terms of appearance and design. Additionally, it is a frequent requirement that an employee needs to wear multiple PPEs simultaneously (e.g., hardhat, safety mask, safety glasses and earmuffs). As an alternative for running multiple classifiers or multi-class classifiers, this study aims to assess the usage of object detectors as a more suited approach for the compliance of the head-mounted PPE. Compared to previous studies related to the PPE detection, which separately considered one to maximum four types of head-mounted PPEs (and altogether covered only eight different types of head-mounted PPE)—this and our previous study^7^ together considered twelve different head-mounted PPE types that are in use across a wider range of industries. Additionally, this is the first study that enveloped and directly bencmarked different deep learning object detection architectures to perform their objective and direct comparison on the developed dataset. Compared to our previous classification based study^7^, which is more comprehensive in terms of the number of PPE types considered, in this study we propose an object detection approach to enable more efficient compliance of body regions with multiple PPEs—such as human head.

**Table 1:** Comparative review of related studies on the topic of computer vision-based compliance of PPE (with the focus on studies related to the head-mounted PPE).

Study	Considered head PPEs	Approach	Architectures employed	Considered environment	Dataset (name, number of images, availability)	Metrics
Proposed	Hardhats, Caps, Hair protection, Sunglasses, Safety glasses, Visors, Welding masks, Cloth masks, Surgical masks, N95 masks, Cartridge respirators, Earmuffs	Pose estimation + Head ROI estimation + Object detection	MobileNetV2-SSD Faster R-CNN, YOLOv5	General purpose	Roboflow, PictorPPE, web-mined images 12,682 images N/A	Precision, Recall
Vukicevic et al.^7^	Face mask, Respirator mask, Earmuffs, Welding mask, Visor, Safety glasses, Hardhat, Head cover	Pose estimation + ROI Classification	HigherHRNet + MobileNetV2	General purpose	Roboflow, PictorPPE, web-mined images 15,728 images N/A	Accuracy, Precision, Recall, F1 Score 95%
Chen and Demechi^8^	Hard hat, full-face mask,	Relationships of the pose landmarks and the detected PPE	OpenPose + YOLOv3	Nuclear power station	Internet images, Webcam captured real world images 3808 images N/A	Precision 97.64% Recall 93.11%
Balakreshnan et al.^9^	Safety glasses	Object detection	Microsoft Azure Custom Vision, n.a	Indoor / laboratory conditions	Images made in laboratory conditions 1291 images N/A	Precision, Recall, Average Precision N/A
Wu et al.^10^	Hardhat	Object detection	SSD	Construction engineering	GDUT-HWD 3174 images Public data	Precision, Recall, Average Precision, Mean Average Precision 83.89%
Delhi et al.^11^	Hardhat, Safety jacket	Object detection	YOLOv3	Construction engineering	Manual collection and image scraping online 2509 images Data available upon request	Precision, 96% Recall, 96% F1 score 96%
Tran et al.^12^	Hardhat, shirt, belt, gloves, pants, shoes	Object detection	YOLOv3	Construction engineering / laboratory	Images collected outdoors by IP camera 12,000 images N/A	Precision, Up to 98% Recall, F1 score
Zhafran et al. ^13^	Hardhat, mask, gloves, yellow vest	Object detection	Fast R-CNN	Construction engineering	Images from CCTV camera, 14,512 images, N/A	Precision, ~ 80% Recall, ~ 80% F1 score ~ 80%
Loey et al.^14^	Medical mask	Object detection	YOLOv2	Covid19, public safety	Medical Masks Dataset (682 images), Face Mask Dataset, (853 images), Public	Average Precision, 81%
Nagrath et al.^15^	Medical mask	Object detection + classification	SSD + MobileNetV2	Covid19, public safety	Combination of various open-source datasets and pictures, 5521 images, Available on GitHub	Accuracy, 92.64% Precision, Recall, F1 Score 93%
Zhang et al.^16^	Hardhat	Object detection	YOLOv5	Construction engineering	Video surveillance on construction site, self-collecting on construction site, Internet crawling, 7076 images, Available upon request	Average Precision, Mean Average Precision ~ 96%

The remainder of this paper is structured as follows: In materials and methods section, after describing the considered dataset we provide high-level overview of the proposed procedure, followed by details about the considered deep learning architectures and corresponding training strategies used in this study; In the experiments and results, we describe our evaluation strategy and accuracy metrics used to assess procedure performances, and present results obtained on the developed dataset; In the discussion and conclusion we provide comparative analysis of the considered deep learning architectures with respect to the state of the art on the topic of computer vision-based PPE compliance.

## Materials and methods

### Considered PPE dataset

Dataset used in this study was collected by merging images from public datasets (Roboflow^18^, Pictor PPE^19^), with additional images obtained using crowdsourcing. The majority of data that support the findings of this study are contained in Roboflow^18^ and Pictor-PPE^19^ public datasets, which terms of use and availability are defined by original datasets authors. Portion of data acquired by authors of this study was collected and processed in accordance with the relevant guidelines proposed with the the Declaration of Helsinki—after obtaining ethical approval from Faculty of Medicine, University of Belgrade, Serbia (Approval No. 1322/X-42) and informed consent from all participants.

**Figure 1:**
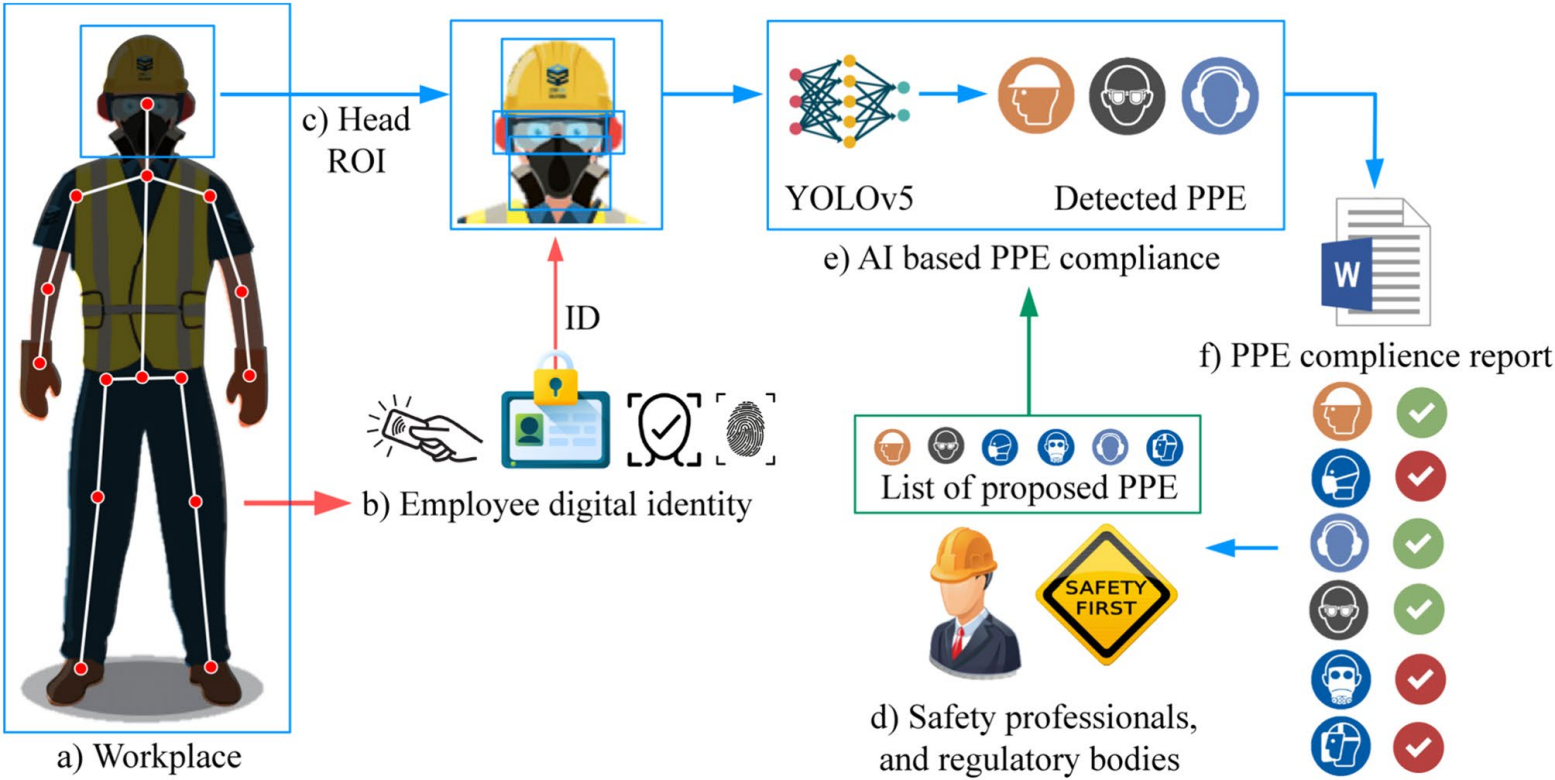
Study overview.

The total number of collected images was 12,682, out of which we annotated 12 various types of PPE. The distribution of PPE classes and structure of the dataset is shown in Table 2. In particular, we studied: (1) hardhats, (2) caps, (3) hair protection caps, (4) sunglasses, (5) safety glasses, (6) visors, (7) welding masks, (8) cloth masks, (9) surgical (medical) masks, (10) N95 masks, (11) cartridge respirators, and 12) earmuffs. To speed-up the image labeling process, we first performed human detection and pose estimation—which helped us to automatically extract regions of interest (ROI) around the human head (Fig. 1a). These ROIs were further cropped and saved as separate image files—which were further labeled and used as input files for the training of deep learning object detectors. The labeling process assumed annotation of bounding boxes around corresponding PPEs’ by using LableMe^19^ and CVAT^20^ software tools.

**Table 2:** Performances of the developed deep learning models for PPE compliance.

PPE category	Number of images	YOLOv5		Faster R-CNN		MobileNet-SSD		Mean		Standard deviation	
		Precision	Recall	Precision	Recall	Precision	Recall	Precision	Recall	Precision	Recall
Hardhats	2552	**1.000**	**0.966**	0.822	0.757	1.000	0.956	0.941	0.893	0.103	0.118
Caps	472	**0.936**	**0.630**	**0.805**	**0.825**	0.929	0.565	0.890	0.673	0.074	0.135
Hair protection	462	1.000	0.274	**0.806**	**0.610**	**0.952**	**0.400**	0.919	0.428	0.101	0.170
Sunglasses	828	1.000	0.711	0.771	0.360	**0.985**	**0.793**	0.919	0.621	0.128	0.230
Safety glasses	2633	0.980	0.923	0.884	0.869	**0.988**	**0.931**	0.951	0.908	0.058	0.034
Visors	1080	0.967	0.547	**0.807**	**0.728**	**0.917**	**0.647**	0.897	0.641	0.082	0.091
Welding masks	431	**0.962**	**0.581**	0.600	0.562	0.867	0.309	0.810	0.484	0.188	0.152
Cloth masks	263	0.595	0.186	**0.727**	**0.333**	0.387	0.224	0.570	0.248	0.171	0.076
Surgical masks	1472	0.993	0.952	0.958	0.797	**1.000**	**0.939**	0.984	0.896	0.023	0.086
N95 masks	457	**1.000**	**0.782**	0.900	0.628	0.937	0.681	0.946	0.697	0.051	0.078
Cartridge respirators	204	0.622	0.137	**0.667**	**0.211**	0.331	0.196	0.540	0.181	0.182	0.039
Earmuffs	1828	0.983	0.643	0.483	0.475	**0.992**	**0.661**	0.819	0.593	0.291	0.103

Mean		0.920	0.611	0.769	0.596	0.857	0.609				
Standard deviation		0.147	0.287	0.133	0.213	0.236	0.275				

Bold values indicate top-performing algorithms for a particular task.

### Procedure overview

Considering sizes of industry halls, it is very difficult to visually observe and check the use of PPE on a company's entire workspace. Instead, we propose the use of AI to automate the PPE compliance at certain checkpoints, as illustrated in Fig. 1. The proposed approach assumes the existence of check-in devices where employees can use a specific electronic certificate (e.g., RFID card). The assumption is that, upon the check-in, the on-site camera could capture the employee image and send it to an AI module consisting of two parts. The first one performs the employee’s pose estimation (Fig. 1a), which assumes detecting body landmark points (e.g., ankles, knees, hips, pelvis, wrists, elbows, shoulders, neck and head)^21^. By using the obtained coordinates for the head and shoulders, the cropped head ROI needs to be forwarded to the second module part—deep learning PPE detector (Fig. 1e). It is assumed that the list of PPEs that need to be used by employees at the particular check-point is defined by the company safety professionals (which are trained to follow recommendations of regulatory bodies). In general, for each check-point, there could be prescribed a different list of proposed PPEs. Therefore, the purpose of AI-driven PPE compliance may be to compare the lists of detected (Fig. 1e) and recommended (Fig. 1d) PPEs and generate the corresponding compliance reports (Fig. 1f). Compared to our previous study^7^, the concept is improved by replacing the multi-class classification module with object detection algorithms: Faster R-CNN, MobileNetV2-SSD and YOLOv5, used to simultaneously inspect various PPEs in frames coming from a camera stream. The pseudocode of the proposed procedure is given in the listing below, while its workflow is illustrated in Fig. 1. The same procedure was used for all considered object detection architectures, where the only adjustment was replacement of the inference function (line 8 in the pseudocode) for a particular model.
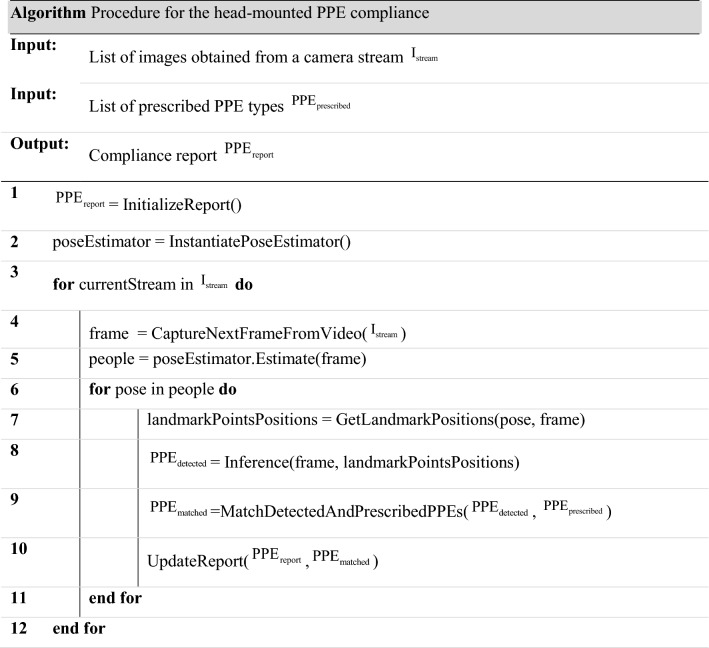


### Considered deep learning architectures

This study considers three types of deep learning architectures: (1) Faster R-CNN, (2) MobileNetV2—SSD, and (3) YOLO, which have been assessed only separately in previous studies on the topic of PPE compliance (see Table 1 for detailed comparative review). The Faster R-CCN is a single-stage network that could be trained in the end-to-end manner^22^. Conceptually, it is composed of a base network for features extraction, region proposal network (RPN)—which generates object proposals (regions with high probability of containing significant objects), and a detection network that generates the final classes and bounding boxes (fully connected layers two of which are common to the classification and the regression layer). Although the Faster R-CCN has been established as accurate and robust architecture (especially when there is a large variance of objects’ sizes in images), its major drawback is the inability to perform in (near) real-time due to the large number of proposals that need to be processed. As an alternative to the RPN, there are single-shot-detectors architectures which directly regress bounding boxes and classes from images. In this study, we considered YOLO and SSD architectures as two most popular regression-based types of object detectors. In particular, we used the MobileNetV2—SSD^15^ architecture which consists of two parts: MobileNetV2^23^ convolutional neural network for the feature map extraction and the Single Shot MultiBox Detector neural network for object detection. This architecture was considered because it has been frequently used for deploying on mobile and edge devices, as it is less demanding in terms of hardware—while it provides a good trade-off between inference spped and accuracy. The third architecture considered in this study was the YOLOv5^24^, which at the moment of this study was the latest version of the YOLO ("You Only Look Once") family. As its’ previous versions, it consists of three parts: (1) convolutional layer for image feature selection/extraction based on the CSPDarknet53^25^, (2) a set of layers based on PANet^26^ for mixing and combining features obtained from convolutional layer, and (3) YOLO layer that predicts classes and bounding boxes based on feature maps collected by the middle layer.

### Training procedure

All models were pretrained on the COCO dataset^27^ and loaded into the PyTorch framework for the transfer learning (which assumes frizzing of base layers, while end layers were trained on the collected datasets to classify PPEs). During the training, we performed the random flip and Gaussian noise online augmentations with the probability of 20%. During the training, each dataset was randomly split into training (70%), validation (15%), and test (15%) datasets. The training was performed using the Adam optimization algorithm^28^. The initial learning rate of the Adam was set to 1e−4, and it was decreased by a factor of 0.1 every 5 epochs. The batch size was 5 for the Faster R-CNN, 10 for the SSD and 16 for the YOLOv5.

## Experiments and results

All the implementations were done by using the Python 3.7.4 programming language; along with the PyTorch 1.6.0 and torchvision 0.7.0 libraries with the cuda 10.2 GPU drivers. All the computations were done on the GPU workstation containing the AMD Threadripper 3970X (32 cores, 3.79 GHz) processor, 128 GB RAM and two Titan RTX (24 GB) + NVLink GPUs.

**Figure 2:**
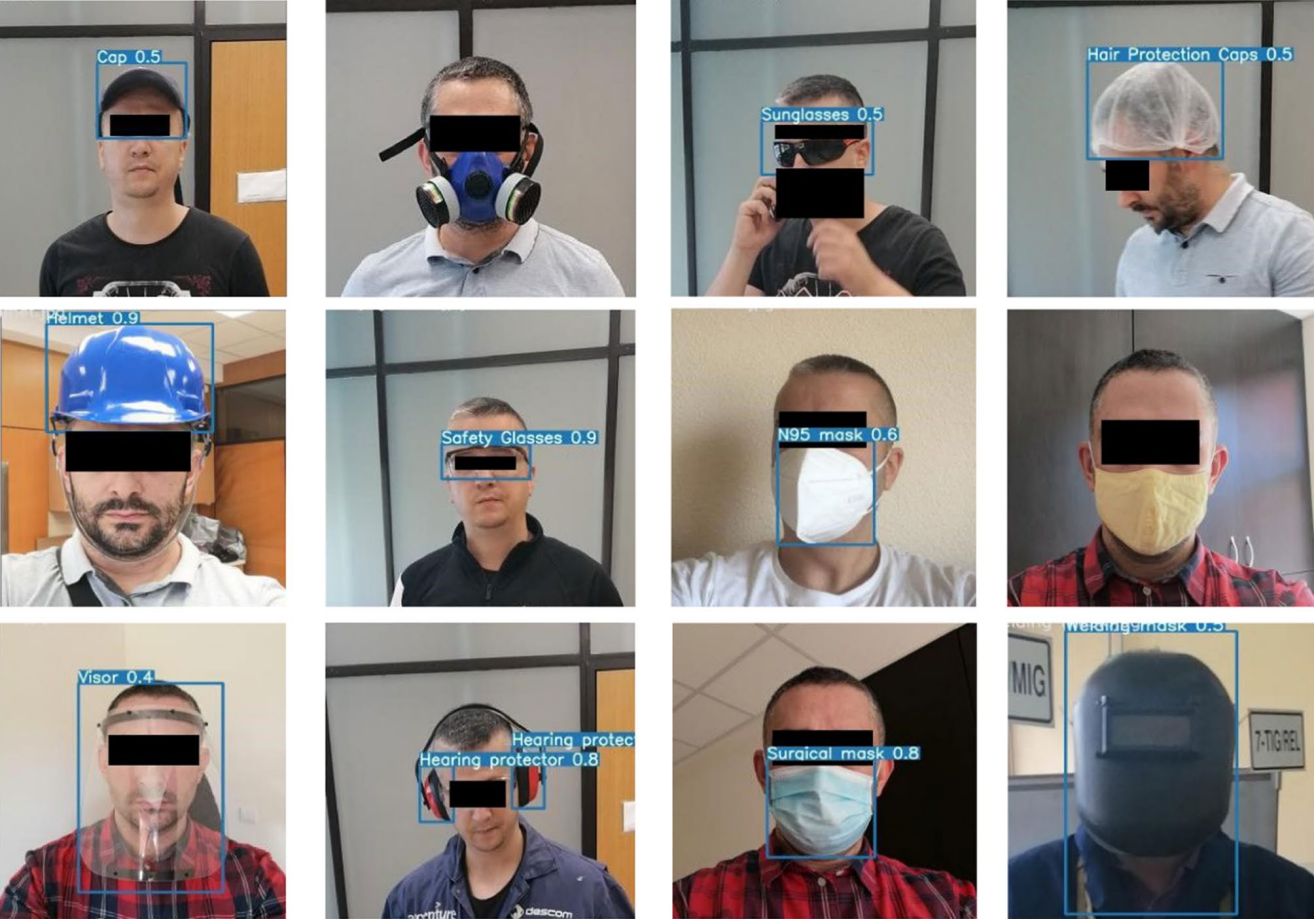
Sample results in laboratory conditions.

The metrics selected for the evaluation and comparison of the developed models included: $Precision=\frac{Tp}{\left(Tp+Fp\right)}$, and $Recall=\frac{Tp}{\left(Tp+Fn\right)}$, where *Tp* are true positive, *Tn* are true negative, *Fp* are false positive, *Fn* are false negative classifications. The obtained results are given in Table 2. Briefly, precision is the ratio of the number of true positive detected objects and the number of positive predictions; Recall represents the ratio of the number of true positive detected objects and the total number of objects. In order to classify the output of the model into one of these three categories, we used the Intersection-over-Union (IoU) metrics^29^. The IoU represents the ratio of intersection area and union area of the ground truth bounding box and a corresponding predicted bounding box around the object detected on an image. By adopting a threshold value of 0.5, we classified the model output as it follows: (1) If the IoU is greater than 0.5, we consider the output to be true positive; (2) If the IoU is greater than of 0.0 and less than 0.5, we consider the output to be false negative; (3) If the IOU is equal to 0.0, i.e. there is a labeled object in the image (ground truth) and the model does not predict it, we consider the output to be false negative. The obtained precision and recall values are shown in Table 2, while sample results are shown in Fig. 2.

In order to benchmark our model with previous studies, we considered two approaches; (1) using public images from the Roboflow^18^ dataset and inference available trained models from literature^7,9,10,19^ (Table 2); (2) using trained models from literature^7^ and assessing them on our data set. Regarding the first scenario, we emphasize that the Robolfow dataset contains only three head-mounted PPE types (hardhat, goggles, and masks)—so the benchmark was restricted only on them (Table 3). Additionally, study^9^ considered only goggles, studies^10^ and^19^ only hardhats, while only the study^7^ considered all three PPEs. We selected the YOLOv5 as the best performing model from Table 2 in terms of mean precision and recall values. The obtained results indicate that the proposed study achieved better performances compared to^7^, while studies^9^ and^10^ achieved slightly better performances on particular PPE types. Regarding the study^7^, we were able to perform direct comparison in 8 out of 12 head-mounted PPE types. The obtained results in Table 4 indicate that classification-based approach in^7^ was better in cases when there were available small data sets, while the object detectors achieved better results on the overall benchmark.

**Table 3:** Comparison of the best developed deep learning model for PPE compliance (based on three types of head-mounted PPEs that exist in the Roboflow public dataset) with models available from the literature on test images from the Roboflow public dataset (P—precision, R—recall).

PPE category	YOLOv5		^7^		^9^		^10^		^19^	
	P	R	P	R	P	R	P	R	P	R
Hardhats	0.922	0.914	0.918	0.913	n/a	n/a	0.891	0.889	0.941	0.918
Safety glasses	0.848	0.820	0.895	0.883	0.921	0.862	n/a	n/a	n/a	n/a
Mask	0.954	0.917	0.911	0.899	n/a	n/a	n/a	n/a	n/a	n/a

**Table 4:** Comparison of the YOLOv5 deep learning model for PPE compliance with our previous classification based model on test images from the dataset used in this paper.

PPE category	YOLOv5		^7^	
	P	R	P	R
Hardhats	1.000	0.966	0.961	0.936
Caps	0.936	0.630	n/a	n/a
Hair protection	1.000	0.274	0.917	0.887
Sunglasses	1.000	0.711	n/a	n/a
Safety glasses	0.980	0.923	0.924	0.919
Visors	0.967	0.547	0.923	0.914
Welding masks	0.962	0.581	0.936	0.908
Cloth masks	0.595	0.186	n/a	n/a
Surgical masks	0.993	0.952	0.920	0.912
N95 masks	1.000	0.782	n/a	n/a
Cartridge respirators	0.622	0.137	0.931	0.894
Earmuffs	0.983	0.643	0.922	0.889

## Discussion

The obtained results in Table 2 indicate that performances of considered detectors varied for different types of head-mounted PPEs. As there are many developed PPE detectors, for the purpose of consistency, we will discuss the trained models with respect to a body part or physiological function that corresponding PPEs protect. For the hearing protection, we considered detection of earmuffs. The top-performing model was the YOLOv5 with the average precision of $0.920\pm 0.147$ and recall $0.611\pm 0.287$, which slightly outperformed the SSD that had precision of $0.857\pm 0.236$ and recall $0.609\pm 0.275$, while Faster R-CNN reached sub-optimal results with average precision of $0.769\pm 0.133$ and recall $0.596\pm 0.213$. For the protection of respiratory system (cartridge respirators, N95 masks, surgical masks, cloth masks,), we report that overall performances of considered architectures were comparable; while for each PPE type different models achieved top-performances. This indicates that there is no gold-standard in terms of selecting the best deep learning architecture for the PPE compliance—instead, we report that one would have to experimentally assess various architectures, and find the most optimal choice for each particular PPE compliance task. Furthermore, by observing the results and datasets size in Table 2, it may be noted that there is a correlation between the number of images present in individual PPE categories and results achieved by different models. For example, all architectures have poor object detection performance for cloth mask and cartridge respirator classes, which is indicated with the mean precision calculated for all three architectures (0.570 and 0.540 for cloth mask and cartridge respirator, respectively). The best performance was obtained by models trained on data from the hardhat and safety glasses—categories with the largest number of image samples. In those two cases the confidence threshold value is over 0.9. In terms of dataset size vs. model accuracy trade-off, it appears that the Faster R-CNN is the most robust on the lack of data, while YOLOv5 most benefit from the data availability. By analyzing the last four columns in Table 1, we report that all architectures achieved good/remarkable performances in the following six categories: hardhat, hair protection, sunglasses, safety glasses, surgical mask, and N95 mask with mean precision greater than 0.9. Satisfactory results were achieved in four other categories: cap, visor, welding mask and earmuffs, with mean precision in range 0.6–0.9. In the remaining two categories, cloth mask and cartridge respirator, the achieved performances may be considered as not satisfactory (mean precision is less than 0.6).

Compared to studies listed in Table 1, this study is the first study that assessed different object detection architectures (studied only separately in literature) to solve the problem of PPE compliance. The proposed study is also the most comprehensive in terms of data/PPEs types and diversity—as we considered the twelve types of head-mounted PPE (which were studied only separately in literature, see Table 1). For the PPE types covered in previous studies (Hard hat, face masks, safety glasses, gloves, medical masks)—we report that we reached state-of-the art performances. We emphasize that the (indirect) comparison with previous studies is avoided in Table 2 because they used different data sets (and cover only a portion of PPEs), while we enveloped all previously considered deep learning architectures—and thus were able to perform their direct and more objective comparison on our own data set.

When it comes to applying such AI-based solutions in real-world industry conditions, we report that a series of challenges and limitations may arise. First, there is an increasing variability of PPE designs and appearance, which nowadays are closer and more difficult to distinguish from the civil equipment (e.g., glasses, earmuffs). Our experimentations on this topic, presented in this and related previous study^7^, indicate that well developed dataset and AI procedures have potential to reduce these problems. Particularly, the ROI classification approach proposed in our previous study^7^ is slightly more robust to PPEs variable—assuming that there is a well-balanced and sufficient amount of labeled data. However, considering the number of different PPEs that may be mounted on a worker head—running the multiclass object detectors is more efficient than running multiple or multi-class classifiers. The major methodological distinction from previous studies in Table 1 is the use of pose estimation algorithms for finding ROIs before applying PPE detectors. The reduction of whole images to head ROIs is significant from two aspects: (1) it eases the collection of images for dataset and (2) it restricts detectors to the particular area of interest (otherwise, for example, it could not distinguish having hardhat in hands and/or on head). Also, from the pose estimation we obtain landmark points for the whole body, not only for the head. Based on those landmarks (hands, feet, torso, etc.) it is possible to crop regions of the images in which there are other parts of the body, where other types of PPE should be used: gloves, safety boots, shoe cover, yellow vest, work suit, etc. Therefore, the solution may be easily upgraded to be useful for inspection of other types of PPE, which have not been the subject of research in this paper.

Regardless of the choice of AI strategy for solving the PPE compliance problem, there are also challenges and limitations related to the computational costs, data privacy and ethical use of such AI solutions in industry practice—which is the subject of our future work. Considering the General Data Protection Regulation (GDPR)^30^, any use of video surveillance for employees monitoring represents a sensitive issue that needs to be justified with appropriate data policies and security measures. In technical terms, conventional video surveillance cameras typically have very wide lenses, and therefore they provide very distorted images. Thus, in order to be able to use conventional surveillance systems for PPE compliance, it is necessary to design it so that in the positions that will be used as checkpoints are high-resolution cameras with narrow lenses. This would make the video surveillance system slightly more expensive, but on the other hand it can significantly contribute to the improvement of working conditions. Finally, processing video streams from a large number of surveillance cameras may be very computationally demanding and financially expensive. As an alternative to the ethical and infrastructural (having sufficient number of cameras, optimal camera positioning etc.) challenges of using conventional surveillance technology—the more promising direction for further development of the technology described in this study is using specialized edge-devices. Having the AI-PPE compliance on the edge device is well suited to be used as automated check points—which may alert employees before entering unsafe areas with inappropriate or without PPE; or even restrict their entry. On the other side, the digitalization of such unsafe moments enables more efficient safety management and exchange of information^31^. Considering the above mentioned, our future work on this topic will be focused on implementing the presented concept on the Edge AI devices – as it should ease the further validation and improvement in real-industry conditions. As the end goal, we aim to envelop various AI modules for recognition of various unsafe acts (which besides PPE compliance may include recognition of unsafe / unergonomic actions)^7,31–36,39–41^, and various manufacturing processes in e.g., logistics^37^ or quality control^38^.

## Conclusion

Ensuring employees' workplace safety within a complex and constantly evolving industrial environment a challenging problem. Since there may be numerous PPE types mounted on various body parts (head, hands, legs, upper body, whole body)—this study was focused on proposing and assessing a solution that could enable automation of the head-mounted PPE compliance. Particularly, we considered three different deep neural network architectures—which were studied only separately in literature. Another distinction from previous studies on the topic is the fact that our approach uses the head ROI estimation before performing the PPE detection—which ensures excluding the false positive cases of detecting PPE on irrelevant image/body regions (e.g., employee holding hardhat or mask in hands). Moreover, we report that the use of automated head ROI detection largely speeds up the collection and labeling of new data, and thus development of new models. For the purpose of validation of the proposed approach, we developed a dataset of 12 distinct PPE types, which makes our study more comprehensive and generic compared to previous ones (which were mainly focused on assessing AI for the PPE compliance of particular PPE types, as shown in Table 1). The results in Table 2 showed that there was no gold standard in terms of selecting the best model—as we found that various deep learning architectures reached different performances for the compliance of various PPE types. This indicates that further studies on this topic should invest more effort into developing more comprehensive datasets that will envelop more different PPE types—as well into the considering and assessing various deep learning architectures in order to objectively find the optimal ones. Instead, we conclude that it is more expected that the proposed and similar solutions will first find its place for the automated PPE compliance at certain checkpoints, e.g., at the entrance of areas where employee authentication may be performed (e.g., by using a RFID) or for the continuous monitoring of PPE misuse into particular zones with high risk from injuries.

## Data Availability

The data that support the findings of this study are contained in Roboflow^18^ and Pictor-PPE^19^ public datasets, which terms of use and availability are defined by original datasets authors. For portion of data collected by authors of this study, we report that they are not publicly available and can-not be distributed as per agreement with the study participants.
